# Clinical Integration of Menin Inhibitors in AML: Evolving Data and Therapeutic Perspectives

**DOI:** 10.32604/or.2025.072443

**Published:** 2026-02-24

**Authors:** Tiffany Chen, Grace Kim, Yekta Rahimi, Monisha Kamdar, Eduardo Fernandez-Hernandez, Karrune Woan, Eric L. Tam, George Yaghmour

**Affiliations:** 1 California University of Science and Medicine, Colton, CA 92324, USA; 2 Keck School of Medicine of USC, University of Southern California, Los Angeles, CA 90033, USA; 3 Department of Medicine, Keck School of Medicine of USC, Los Angeles, CA 90033, USA; 4 Jane Ann Nohl Division of Hematology and Center for the Study of Blood Disease, USC Norris Comprehensive Cancer Center, Los Angeles, CA 90033, USA

**Keywords:** Menin, menin inhibitor, acute myeloid leukemia (AML), leukemia, revumenib, ziftomenib, bleximenib, icovamenib

## Abstract

Acute myeloid leukemia (AML) remains a biologically heterogeneous disease with historically limited targeted therapies and poor outcomes. The development of menin inhibitors represents a promising shift, particularly for patients harboring *KMT2A* rearrangements (*KMT2A*r) and *NPM1* mutations (*NPM1*m). This manuscript reviews the molecular rationale of menin inhibition for aberrant homeobox/myeloid ectopic insertion site 1 (HOX/MEIS1)-driven gene expression and leukemogenesis, clinical trial outcomes, and safety data for menin inhibitors, with a focus on recently FDA-approved revumenib and several other agents in development, ziftomenib (KO-539), bleximenib (JNJ-75276617), and icovamenib (BMF-219). We also focused our discussion on future directions to include resistance mechanisms, biomarker identification and monitoring strategies, and combination therapies. Menin inhibition is now being clinically integrated into relapsed/refractory and frontline treatment settings.

## 1 Introduction

Acute myeloid leukemia (AML) is a clonal hematologic malignancy characterized by uncontrolled proliferation of myeloid precursor cells in the bone marrow, leading to improper hematopoietic differentiation and subsequent cytopenias [1,2]. Survival expectations remain age-dependent, with a 62% estimated 5-year survival in patients diagnosed under the age of 50 years, 37% survival for patients 50–64 years of age, and 9.4% for patients 65 years and older at diagnosis [1]. Outcomes are especially poor for relapsed or refractory (R/R) AML, for elderly patients unfit for intensive therapy, and for those with adverse cytogenetic or molecular features [3]. The pathogenesis of AML is driven by a myriad of genetic and/or epigenetic abnormalities [4,5]. Over the past decade, emerging treatments, such as FMS-like tyrosine kinase 3 (FLT3) and isocitrate dehydrogenase 1/isocitrate dehydrogenase 2 (IDH1/IDH2) inhibitors, have focused on targeting molecularly defined AML subgroups, which have improved overall survival (OS) in patients with select mutations. However, the OS rates of AML patients with histone-lysine N-methyltransferase 2A rearrangements (*KMT2A*r) are still poor, with a median OS of as low as 2.4 months [6]. One promising therapeutic avenue for these patients is menin inhibition, which disrupts the interaction between KMT2A and the nuclear scaffold protein, menin, that is a key mediator in leukemogenic transcriptional activation. Thus far, menin inhibitors have shown clinical efficacy with high rates of minimal residual disease (MRD) negativity, which has led to regulatory approval for one menin inhibitor, revumenib, in the treatment of R/R AML in adult and pediatric patients with *KMT2A*r and nucleophosmin 1 mutation (*NPM1*m) [7]. Their specificity and ability to redirect leukemic proliferation toward differentiation distinguish menin inhibitors from conventional cytotoxic agents, establishing them as a novel class of targeted therapies in the treatment of AML. Menin inhibitors may also complement other targeted AML therapies, including FLT3, IDH1/IDH2, and B-cell lymphoma/leukemia 2 (BCL2) inhibitors, particularly since many patients can possess co-mutations and relapse after initial treatment [1,8]. In this review, we will examine the development of menin inhibitors, summarize the latest clinical advances, identify biomarker testing, and discuss the challenges and future directions in leveraging this novel therapeutic strategy to improve outcomes for patients with AML.

## 2 Pathophysiology and Rationale of Menin Inhibitors

During normal hematopoiesis, the menin-KMT2A interaction plays a crucial role in regulating chromatin modification and gene expression [9,10]. Menin, encoded by menin 1 (*MEN1)* on chromosome 11q13, functions as a nuclear scaffold protein that binds to the N-terminal region of KMT2A via two highly conserved menin-binding motifs (MBM1 and MBM2) [11,12]. This association directs KMT2A to its target gene promoters, where its histone H3 lysine 4 (H3K4) methyltransferase activity promotes a transcriptionally active chromatin state. The menin-KMT2A complex targets many genes essential for hematopoietic stem cell (HSC) maintenance, such as homeobox A9 (HOXA9) and MEIS1, and dysfunction in their interaction has shown impaired adaptive stress responses and self-renewal in HSCs [13]. This dependence on menin is maintained in *KMT2A*r AML as well, rendering menin as a possible target for inhibition.

*KMT2A* rearrangements occur in roughly 5%–10% of adult AML cases and 20% of *de novo* cases in children [8,14,15]. Over 130 different fusion partners have been described, but only a handful of them account for the majority of *KMT2A*r acute leukemia cases, including the transcriptional cofactors ALL-1 fused gene from chromosome 4 (AF4: ~36%), ALL-1 fused gene from chromosome 9 (AF9: ~19%), and eleven-nineteen leukemia (ENL: ~13%) [8,16,17]. These fusion proteins lose the KMT2A C-terminal Su(var)3-9/Enhancer of Zeste/Trithorax (SET) domain responsible for H3K4 methyltransferase activity but retain the N-terminus that binds to menin [16,18]. Thus, upon binding to menin, the *KMT2A*r fusion proteins translocate to the nucleus, where they constitutively recruit transcriptional coactivators and histone acetyltransferases at target promoter sites. This leads to aberrant expression of genes involved in HSC proliferation, most notably the *HOX* family genes and their cofactor, *MEIS1* [10,16,19–21]. Constitutive activation of the HOX/MEIS1 complex prevents their epigenetic repression and subsequently drives a differentiation block in precursor cells. Different mechanisms of how aberrant gene expression is achieved by *KMT2A*r variants have been proposed, yet there is a shared dependence on menin for nuclear localization.

Other genetic abnormalities, including *NPM1*m and nucleoporin 98-rearranged (*NUP98*r) AML, rely on menin interaction with KMT2A to maintain their leukemogenic states. *NPM1*m AML represents 25%–30% of adult cases [22], while *NUP98*r AML is frequently reported in pediatric populations (4%–7%) [23,24], though a recent study showed a relatively high prevalence in adult East Asian patients [25]. The exact pathophysiology of *NPM1*m AML remains elusive, however past studies suggest that NPM1m plays a role at both the nuclear and cytoplasmic levels, driving constitutive *HOX*/*MEIS1* expression and preventing apoptotic protease activity, respectively. NPM1m is recruited to chromatin by the transport protein, exoportin 1 (XPO1), and localizes at sites that are already enriched with menin-*KMT2A*, most notably *HOX*/*MEIS* [26–28]. It is unclear how NPM1m promotes HOX/MEIS transcription, however, degradation of NPM1m resulted in a significant reduction of *HOX*/*MEIS* expression in *in vitro* studies [27,29]. In addition, inhibition of menin-KMT2A interaction prevented not only KMT2A binding to their target loci but also NPM1m to the same loci, including *MEIS1*, runt-related transcription factor 2 (*RUNX2)*, and others [30]. Similarly, NUP98r fusion proteins directly interact with menin-KMT2A to drive leukemogenesis via upregulation of HOX/MEIS1. Disruption of the menin-KMT2A interaction by menin inhibitors led to the displacement of KMT2A and NUP98 fusion proteins from chromatin at proleukemogenic genes in both *in vitro* and patient-derived xenograft models [31–33].

**Figure 1:**
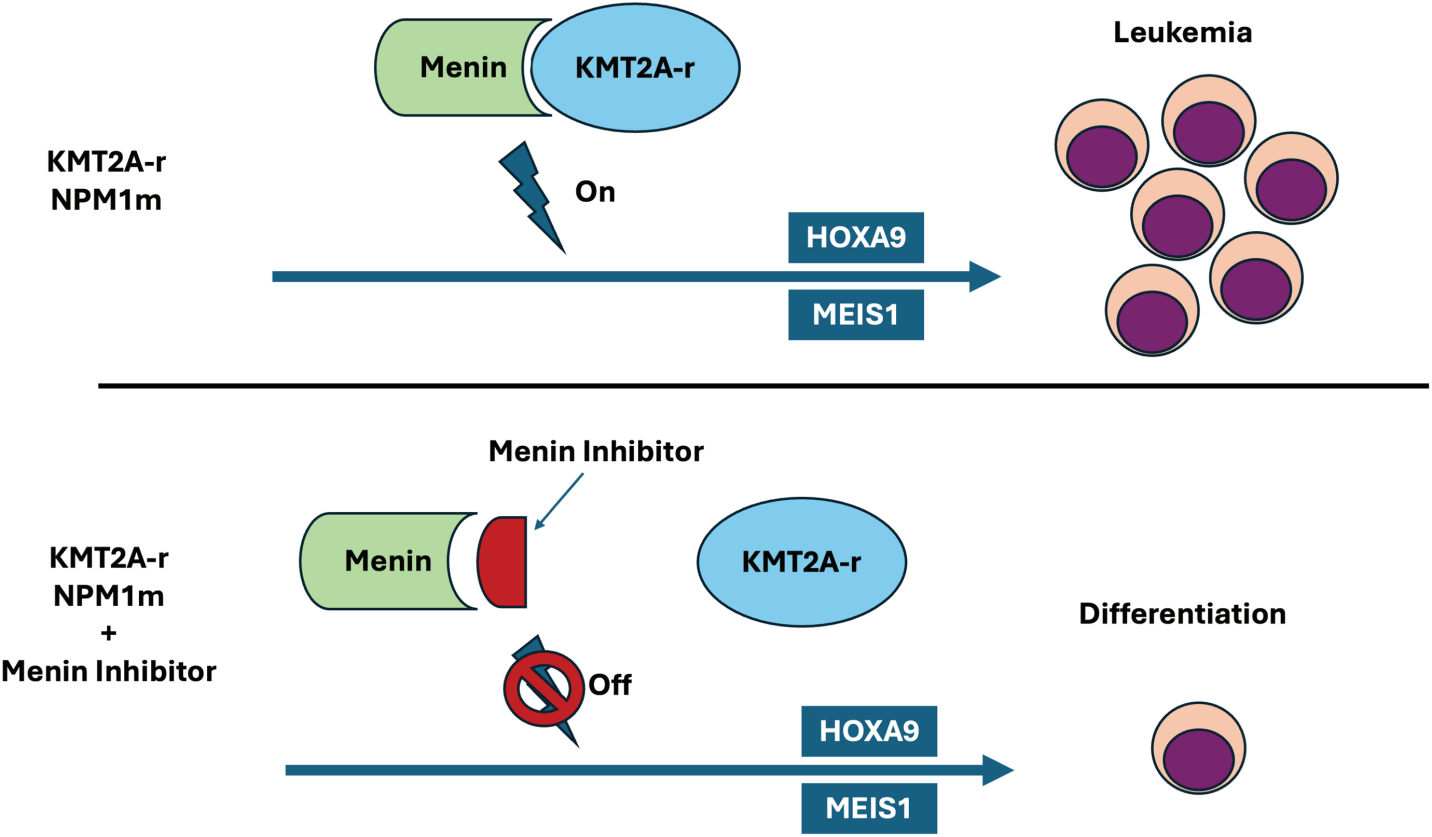
Menin binds to KMT2A and is an essential cofactor for interactions with *HOX* gene promoters. *KMT2A*r leukemias are characterized by the abnormal overexpression of *HOX* genes and their cofactor, *MEIS1*. *NPM1*m is primarily located in the cytoplasm and upregulates *HOX* genes. This results in a block of hematopoietic differentiation and contributes to leukemic transformation. Menin inhibitors disrupt the chromatin complex between menin and KMT2A and inhibit this interaction, thereby disrupting the abnormal transcriptional program leading to leukemogenesis. Original graphic, Microsoft PowerPoint Version 16.103.1

These findings have led to the development of small-molecule menin inhibitors that aim to disrupt the menin–KMT2A interaction, aiming to extinguish the HOX/MEIS1 transcriptional program underlying these leukemias (Fig. 1). Preclinical studies have repeatedly shown that small-molecule menin inhibitors, including revumenib, ziftomenib (KO-539), bleximenib (JNJ-75276617), and icovamenib (BMF-219), can effectively disrupt the menin–KMT2A interaction. They reduce target gene expression, promote differentiation and apoptosis, and suppress AML growth in cell lines and patient-derived xenografts across *KMT2A*r, *NPM1*m, and *NUP98*r models [34]. Revumenib and ziftomenib have been developed to bind to the menin binding pocket with high affinity and prevent the interaction with the KMT2A fusion proteins [35,36].

## 3 Menin Inhibitors Approved and in Clinical Trials

Currently, the menin inhibitors under evaluation in clinical trials include revumenib, ziftomenib, BN104, bleximenib, icovamenib (BMF-219), and enzomenib (Tables 1 and A1). Preliminary results are available for trials with revumenib, ziftomenib, BN104, bleximenib, icovamenib, and enzomenib in monotherapy and combination therapy regimens (Table 2).

**Table 1:** Menin inhibitor clinical trials

Drug	Regimen	Phase	Diagnosis	Mutation
Revumenib (SNDX-5613)	Monotherapy	Phase 1 (NCT06575296)	AML, ALL (post-allogeneic HSCT)	*KMT2A, NPM1*
		Phase 1/2 (NCT04065399)	AML, ALL	*KMT2A, NPM1, NUP98*
		Phase 2 (NCT06229912)	AML, ALL	*KMT2A, NPM1, NUP98, NUP214* (upregulation of HOX genes)
	Combination	Phase 1 (NCT06222580)	AML	*KMT2A, NPM1, FLT3*
		Phase 1 (NCT06313437)	AML	*NPM1, FLT3*
		Phase 1 (NCT05886049)	AML	*KMT2A, NPM1*
		Phase 1 (NCT06177067)	AML, ALAL	*KMT2A, NPM1, NUP98, NUP214*
		Phase 1 (NCT07052994)	AML, MPAL	*KMT2, NPM1, NUP98, UBTF-ITD*
		Phase 1 (NCT06226571)	AML	*KMT2, NPM1, NUP98*
		Phase 1/2 (NCT05360160)	AML	*KMT2A, NPM1, NUP98*
		Phase 1/2 (NCT06284486)	AML	*KMT2A, NPM1, NUP98*
		Phase 2 (NCT05761171)	ALL	*KMT2A*
		Phase 3 (NCT06652438)	AML	*KMT2A, NPM1*
		Phase 3 (NCT07211958)	AML	*NPM1*
Ziftomenib	Monotherapy	KO-MEN-001 Phase 1/2 (NCT04067336)	AML	*KMT2A, NPM1*
		Phase 2 (NCT06930352)	AML (not eligible for standard therapy)	*KMT2A, NPM1*
	Combination	KO-MEN-007 Phase 1 (NCT05735184)	AML	*KMT2A, NPM1*
		Phase 1 (NCT06448013)	AML, MPAL	*KMT2A, NPM1, NUP98, UBTF-ITD*
		Phase 1 (NCT06376162)	AML	*KMT2A, NPM1, NUP98*
		Phase 1 (NCT06397027)	AML, MPAL	*KMT2A, NPM1, NUP98*
		Phase 1 (NCT06769490)	AML, MPAL	*KMT2A, NPM1, NUP98*
		Phase 1 (NCT06001788)	AML	*KMT2A, NPM1, FLT3*
		Phase 3 (NCT07007312)	AML	*KMT2A, NPM1, FLT3*
BN104	Monotherapy	Phase 1/2 (NCT06052813)	AML, ALL	*KMT2A, NPM1*
	Combination	Phase 1/2 (NCT06746519)	AML	*KMT2A, NPM1, NUP98*
Bleximenib (JNJ-75276617)	Monotherapy	Phase 1/2 (NCT04811560)	AML, ALL	Phase 1: *KMT2A, NPM1, NUP98, NUP214*; Phase 2: *KMT2A, NPM1*
	Combination	Phase 1 (NCT05453903)	AML	*KMT2A, NPM1, NUP98, NUP214*
		Phase 3 (NCT06852222)	AML	*KMT2A, NPM1*
Icovamenib (BMF-219)	Monotherapy	COVALENT-101 Phase 1 (NCT05153330)	AML, ALL, DLBCL, MM, CLL/SLL	*KMT2A/MLL1, NPM1*
Enzomenib (DSP-5336)	Monotherapy & Combination	Phase 1/2 (NCT04988555)	AML, ALL, MM, MDS	*MLL1, NPM1*

Note: All up-to-date clinical trial information can be found on clinicaltrials.gov. Abbreviations: AML, acute myeloid leukemia; ALL, acute lymphoblastic leukemia; HSCT, hematopoietic stem cell transplantation; ALAL, acute leukemia of ambiguous lineage; MPAL, mixed phenotype acute leukemia; DLBCL, diffuse large B-cell lymphoma; MM, multiple myeloma; CLL, chronic lymphocytic leukemia; SLL, small lymphocytic lymphoma; MDS, myelodysplastic syndrome.

**Table 2:** Interim data from active menin inhibitor clinical trials

Drug	Trial/NCT	Regimen	Patient characteristic	ORR	CR/CRh	Differentiation syndrome	QTc Prolongation	Grade ≥ 3 TEAE	Most common TEAE	DLT
Revumenib [37,38]	AUGMENT-101 (NCT04065399)	Monotherapy	n = 116 (*KMT2A*r = 116)	64% (62/97)	23% (22/97)	26.7% (Grade 3 = 51.6%, Grade 4 = 3.2%)	29.3% (Grade 3 = 44.1%)	Febrile neutropenia (39%), anemia (20%), platelet count decreased (16%), neutropenia (15%), differentiation syndrome (15%), sepsis (14%), QTc prolongation (13%)	Nausea (44.8%), febrile neutropenia (39.7%), vomiting (34.5%), diarrhea (30.2%), QTc prolongation (29.3%)	N/A
			n = 84 (*NPM1*m = 84) [39]	46.9% (30/64)	23.4% (15/64)	19.0% (Grade 3 = 10.7%, Grade 4 = 2.4%)	42.9% (Grade 3 = 20.2%, Grade 4 = 2.4%)	Febrile neutropenia (33.3%), anemia (25%), QTc prolongation (22.6%), platelet count decreased (16.7%), sepsis (15.5%), differentiation syndrome (13.1%)	QTc prolongation (42.9%), vomiting (36.9%), febrile neutropenia (34.5%), hypokalemia (32.1%), differentiation syndrome (19.0%)	N/A
			n = 34 (*NUP98*r = 5) [40]	N/A	20% (1/5)	2.9% (Grade 3 = 2.9%)	15% (Grade 3 = 2.9%)	Anemia (9%), neutrophil count decreased (9%), differentiation syndrome (2.9%), QTc prolongation (2.9%)	Nausea (18%), dysgeusia (15%), QTc prolongation (15%)	N/A
	SAVE (NCT05360160)	Combination: Decitabine/ Cedazuridine (ASTX727) + Venetoclax	n = 33 (*KMT2A*r = 16, *NPM1*m = 12, *NUP98*r = 5) [38]	82% (*KMT2A*r = 88%, *NPM1*m = 67%, *NUP98*r = 100%)	48% (*KMT2A*r = 44%, *NPM1*m = 50%, *NUP98*r = 60%)	All grade = 9%, Grade ≥ 3 = 3%	All grade = 64%, Grade ≥ 3 = 9%	Febrile neutropenia (33%), lung infection (33%), sepsis (18%)	QTc prolongation (64%), elevated AST/ALT (58%), nausea (55%)	Thrombocytopenia
	BEAT-AML (NCT03013998)	Combination: Venetoclax/Azacitidine	n = 43 (*KMT2A*r = 9, *NPM1*m = 34) [41]	88.4% (38/43)	69.7% (30/43)	19% (Grade 3 = 25%)	44% (Grade 3 = 26%)	Febrile neutropenia (26%), acute kidney injury (19%), dyspnea 14%), QTc prolongation (12%), hypokalemia (12%), differentiation syndrome (5%)	Nausea (60%), constipation (53%), QTc prolongation (44%), hypokalemia (44%), differentiation syndrome (19%)	Thrombocytopenia
Ziftomenib	KOMET-001 (NCT04067336)	Phase 1 Monotherapy	n = 83 (*KMT2A*r = 39, *NPM1*m = 28) [42]	*NPM1*m = 45%	25% (9/36)	22% (Grade ≥ 3 = 67%)	None	Anemia (24%), febrile neutropenia (22%), pneumonia (19%), differentiation syndrome (15%), thrombocytopenia (13%), sepsis (12%)	Diarrhea (31%), nausea (29%), anemia (25%), febrile neutropenia (23%), hypokalemia (23%), differentiation syndrome (22%)	Differentiation syndrome and pneumonitis
		Phase 2 Monotherapy	n = 92 (*NPM1*m = 92) [43]	33% (30/92)	22% (20/92)	25% (Grade 3 = 61%)	All grade = 3%, Grade 3 = 2%	Febrile neutropenia (26%), anemia (20%), thrombocytopenia (20%), differentiation syndrome (15%)	Diarrhea (28%), febrile neutropenia (26%), differentiation syndrome (25%), nausea (25%)	N/A
	KOMET-007 (NCT05735184)	Combination: Venetoclax/Azacitidine	n = 34 (*KMT2A*r = 20, *NPM1*m = 14) [44]	*NPM1*m (200mg) = 100%, *NPM1*m (400mg) = 67%, *KMT2A*r (200mg) = 43%, *KMT2A*r (400mg) = 33%	CRc = 80% *NPM1*m (200mg), 50% *NPM1*m (400mg), 29% *KMT2A*r (200mg), 17% *KMT2A*r (400mg)	12% (Grade 3 = 75%)	None	Febrile neutropenia (35%), platelet count decreased (35%), anemia (26%), decreased neutrophil count (24%), pneumonia (24%)	N/A	None
		Combination: Cytarabine/Daunorubicin (7+3)	n = 34 (*KMT2A*r = 19, *NPM1*m = 15) [45]	N/A	CRc = 100% *NPM1*m (200mg), 86% *NPM1*m (400mg), 90% *KMT2A*r (200mg), 63% *KMT2A*r (400mg)	None	None	Febrile neutropenia (56%), platelet count decreased (47%), decreased neutrophil count (38%), anemia (32%), decreased WBC count (29%)	N/A	None
BN104 [46]	NCT06052813	Monotherapy	n = 20 (*KMT2A*r = 12, *NPM1*m = 5, *NUP98*r = 2, *KMT2A*r + *NPM1*m = 1)	88.9% (8/9)	33.3% (3/9)	Grade 2 = 10%	Grade 1 = 10%	Febrile neutropenia (15%) and pneumonia (10%)	Vomiting (35%) and nausea (30%)	None
Bleximenib [47,48]	NCT04811560	Monotherapy	n = 121 (*KMT2A*r = 73, *NPM1*m = 48) [47]	45mg BID = 39% (5/13), 90/100mg BID = 50% (10/20), 150mg BID = 50% (10/20)	45mg BID = 23% (3/13), 90/100mg BID = 35% (7/20), 150mg BID = 30% (6/20)	All grade = 14%, Grade ≥ 3 = 7%	0.8% (1/121)	Neutropenia (11%), thrombocytopenia (8%), differentiation syndrome (7%)	Differentiation syndrome (13%), neutropenia (12%), thrombocytopenia (11%), nausea (9%)	Neutropenia and QTc prolongation
	NCT05453903	Combination: Cytarabine/Daunorubicin (7+3)	n = 22 (*KMT2A*r = 11, *NPM1*m = 11) [48]	93% (*KMT2A*r = 83%, *NPM1*m = 100%)	CR = 79%, CR/CRh = 86% (CR/CRh: *KMT2A*r = 83%, *NPM1*m = 88%)	None	None attributed to bleximenib	Febrile neutropenia (64%), thrombocytopenia (68%), anemia (41%), neutropenia (41%)	Diarrhea (77%), thrombocytopenia (68%), febrile neutropenia (64%)	None
		Combination: Venetoclax/Azacitidine	n = 120 (*KMT2A*r = 52, *NPM1*m = 68) [49]	50mg R/R = 76%, 100mg R/R = 79%, 50mg ND = 77%, 100mg ND = 92%	50mg R/R CRc = 32%, 100mg R/R CRc = 54%, 50mg ND CRc = 62%, 100mg ND CRc = 85%	4% (Grade 2–3 = 4, Grade 4 = 1)	None	Thrombocytopenia (53%), anemia (48%), neutropenia (46%)	Nausea (60%), thrombocytopenia (55%), anemia (51%)	Differentiation syndrome and diverticulitis
BMF-219 [50]	NCT05153330	Monotherapy	n = 26 (*KMT2A*r = 6, *NPM1*m = 4)	N/A	CR = 20% (1/5), CRi = 20% (1/5)	Grade ≥ 3 = 13%	None	Differentiation syndrome (13%)	Vomiting (13%), differentiation syndrome (13%)	None
Enzomenib [51]	NCT04988555	Monotherapy	n = 84 [51]	62.5% (25/40)	37.5% (15/40)	10.70%	Grade 2 = 2.5%, Grade 1 = 2.5%	N/A	Nausea (39.5%), vomiting (29.6%), febrile neutropenia (22.2%)	None

Note: ORR, overall response rate; CR, complete remission; CRh, complete remission with partial hematologic recovery; CRi, complete remission with incomplete count recovery; QTc, corrected QT interval; TEAE, treatment emergent adverse event; DLT, dose limiting toxicity; AST, aspartate aminotransferase; ALT, alanine aminotransferase; R/R, relapsed or refractory; ND, newly diagnosed; N/A, not applicable.

### 3.1 Revumenib

Revumenib is the first FDA-approved menin inhibitor for R/R AML with *KMT2A* translocations. Approval was granted on 15 November 2024, for ages 1 year and older, and indication for *NPM1*m AML was approved on 24 October 2025 [52]. AUGMENT-101 is a phase I/II clinical trial that studied the safety and efficacy of revumenib in patients with R/R *KMT2A*r AML and R/R *NPM1*m AML [39]. This study reported an updated complete remission (CR) and CR with partial hematologic recovery (CRh) rate of 23% and overall response rate (ORR) of 64% in the efficacy population of patients with *KMT2A*r AML. Additionally, MRD negative status was achieved in 58% of the evaluable patients in the CR + CRh cohort and 34% of responders continued forward to receive an allogeneic hematopoietic stem cell transplantation (HSCT). Adverse events of grade 3 or higher (91%) included febrile neutropenia (39%), anemia (20%), thrombocytopenia (16%), differentiation syndrome (15%), neutropenia (15%), leukopenia (15%), sepsis (14%), and corrected QT interval (QTc) prolongation (13%) [37]. Dose adjustment for QTc prolongation and treatment with corticosteroids and hydroxyurea for differentiation syndrome was effective and resulted in no discontinuation of treatment with revumenib [53]. In the study arm for R/R *NPM1*m AML, the CR + CRh rate was 23.4% and ORR was 46.9% with 16.7% of responders proceeding to allogeneic HSCT. Similar to the *KMT2A*r cohort, 91.7% of patients experienced grade 3 or higher adverse events such as febrile neutropenia (33.3%), anemia (25.0%), QTc prolongation (22.6%), sepsis (15.5%), and differentiation syndrome (13.1%). Of note, there is a higher incidence of QTc prolongation in the R/R *NPM1*m AML study arm of AUGMENT-101. Differentiation syndrome and QTc prolongation resulted in discontinuation in 2 patients [39]. In short, revumenib offers clinical benefit for patients with R/R *KMT2A*r and *NPM1*m AML, enabling those in remission to pursue allogeneic HSCT, while displaying a manageable and predictable safety profile (Tables 3 and 4).

**Table 3:** Summary of Revumenib Phase 2 outcomes in *KMT2A*r and *NPM1*m AML

Patient subset	ORR (%)	CRc (%)	Median OS (Months)	MRD-Negative Rate (%)
*KMT2A*r (n = 57)	63%	39%	8.0	82% (CRc subgroup)
*NPM1*m (n = 64)	~50%	36%	23.3 (CR + CRh)	Not reported

Note: ORR, overall response rate; CR, complete remission; CRc, composite complete remission; CRh, complete remission with partial hematologic recovery; OS, overall survival; MRD, measurable residual disease.

**Table 4:** Summary of Revumenib safety profile (Phase 2)

Adverse event	Incidence (Any grade)	Grade ≥3 Incidence
Differentiation syndrome	19%	10.7% (Grade 3), 2.4% (Grade 4)
QTc Prolongation	42.9%	20.2% (Grade 3), 2.4% (Grade 4)
Cytopenias (Combined)	≥25%	≥10%

Note: QTc, corrected QT interval.

In addition to patients with *KMT2A*r and *NPM1*m AML, the AUGMENT-101 trial enrolled 34 patients without either mutation [40]. Among these patients, 5 had R/R NUP98r AML. Upon revumenib treatment, 60% achieved morphological remission. MRD-negative status was attained in 40% of patients and 20% continued to undergo hematopoietic stem cell transplant. Safety data for the cohort of 34 patients demonstrated 24% of patients experiencing grade 3 or higher adverse events, including anemia (9%), neutropenia (9%), differentiation syndrome (2.9%), and QTc prolongation (2.9%). Overall, the safety profile and efficacy of treatment with revumenib in *NUP98*r AML patients is promising. Future studies are currently underway to evaluate the efficacy of revumenib in treating AML associated with HOX upregulation, pointing towards a promising expansion of the therapeutic application of menin inhibitors [40].

Revumenib is also proven efficacious in combination therapy settings. The SAVE clinical trial is currently exploring the utilization of revumenib in combination with venetoclax and hypomethylating agent ASTX727 (decitabine/cedazuridine) in R/R KMT2Ar, NPM1m, and NUP98r AML. The study reported a CR + CRh rate of 48% (KMT2Ar: 44%, NPM1m: 50%, NUP98r: 60%) and ORR of 82% (KMT2Ar: 88%, NPM1m: 67%, NUP98r: 100%). QTc prolongation was reported in 64% of patients (Grade 3: 6%, Grade 4: 3%) and differentiation syndrome occurred in 9% (Grade 3: 3%), with no notable differences in safety. It is important to mention that MRD negativity was 88% with 39% of patients proceeding with allogeneic HSCT [38]. The high response rates and high rate of MRD negativity reinforce the potential of revumenib to be highly effective in combination therapy for R/R AML.

Revumenib is also under investigation in the BEAT AML trial in combination with azacitidine and venetoclax (ven/aza) for newly diagnosed (ND) KMT2Ar or NPM1m AML patients over 60 years of age. Interim analysis demonstrated the ORR was 88.4% (NPM1m: 85.3%, KMT2Ar: 100%), CR + CRh + complete remission with incomplete count recovery (CRi) rate was 81.4% (NPM1m: 79.4%, KMT2Ar: 88.9%), and CR rate was 67.4% (NPM1m: 64.7%, KMT2Ar: 77.8%) for the 43 patients treated in this study. It is important to note that no patients had refractory disease and all 37 patients with MRD assessments had MRD negativity after treatment. Adverse events were comparable to other studies with revumenib, including nausea (60%), QTc prolongation (44%), hypokalemia (44%), and differentiation syndrome (19%). Neither QTc prolongation nor differentiation syndrome resulted in discontinuation of revumenib in this study [41]. Overall, the BEAT AML study demonstrates that the combination therapy of revumenib, azacitidine, and venetoclax is a safe and suitable option for older individuals with newly diagnosed *KMT2A*r or *NPM1*m AML, as it yields high response rates, including MRD negativity. The efficacy demonstrated in this study warrants further investigation of the safety and efficacy of revumenib with 7+3 intensive chemotherapy in patients with newly diagnosed *KMT2A*r, *NPM1*m, or *NUP98*r AML patients (NCT06226571) and comparison of revumenib combined with azacitidine and venetoclax against a control group receiving placebo with aza/ven (NCT06652438) (EHA Library, Abstract PB2576).

Revumenib has demonstrated efficacy across multiple settings in both newly diagnosed and relapsed/refractory AML, both as monotherapy and in combination. While its efficacy is clear, an increased incidence of QTc prolongation requires careful monitoring by clinicians. The role of revumenib in the therapeutic landscape will depend on how the risk-benefit profile compares with the other emerging menin inhibitors and the current standard of care treatments.

### 3.2 Ziftomenib

Ziftomenib is being studied as both monotherapy in AML and in various combination therapy approaches. The KOMET-001 study, a phase 1 trial of ziftomenib as a monotherapy for R/R *KMT2A*r and *NPM1*m AML, reported an overall CR + CRh rate of 25%. Patients with *NPM1* mutations had a CR rate of 35%, ORR of 45%, and an MRD negativity rate of 67% in the six patients assessed. Adverse events of grade 3 or higher in this study included anemia (24%), febrile neutropenia (22%), pneumonia (19%), differentiation syndrome (15%), thrombocytopenia (13%), and sepsis (12%). A notable observation from the KOMET-001 study was that *KMT2A*r AML patients had higher occurrences and severity of differentiation syndrome compared to *NPM1*m AML patients, leading to the discontinuation of *KMT2A*r AML patient enrollment [42]. Phase 2 of KOMET-001 focused on patients with R/R NPM1m AML who received ziftomenib 600 mg daily. Patients achieved a CR + CRh rate of 22%, ORR of 33%, and MRD negativity rate of 61%. The safety profile was similar to Phase 1 with grade 3 adverse events including febrile neutropenia (26%), anemia (20%), thrombocytopenia (20%), differentiation syndrome (15%) with 2 patients discontinuing treatment, pneumonia (14%), sepsis (14%), hypokalemia (13%), and QTc prolongation (2%) [43]. The KOMET-007 study focuses on the use of ziftomenib in combination with standard chemotherapy. Two arms of this ongoing study include ziftomenib with venetoclax and azacitidine (ven/aza) in R/R *KMT2A*r and *NPM1*m AML and ziftomenib with cytarabine and daunorubicin (7+3) in newly diagnosed *KMT2A*r and *NPM1*m AML. The interim data for ziftomenib combined with venetoclax + azacitidine at a dose of 200 mg reported a composite complete remission (CRc) rate of 80% in *NPM1*m AML and 29% in *KMT2A*r, while a dose of 400 mg resulted in a CRc rate of 50% in *NPM1*m AML and 17% in *KMT2A*r AML. Differentiation syndrome was present in 12% of the treated patients, with most cases occurring in patients with *KMT2A*r AML. Differentiation syndrome was manageable, and enrollment of *KMT2A*r AML patients is ongoing [44]. The ziftomenib with 7+3 arm reports a CRc rate of 100% at 200 mg and 86% at 400 mg in *NPM1*m AML patients and a CRc rate of 90% at 200 mg and 63% at 400 mg in *KMT2A*r AML patients [45]. The updated results of ziftomenib at 600 mg with 7+3 reports a CRc rate of 94% in *NPM1*m patients and 83% in *KMT2A*r patients [45]. Ziftomenib with 7+3 is generally well tolerated with no reported cases of differentiation syndrome and QTc prolongation. Ziftomenib in combination with venetoclax + azacitidine or 7+3 presented with similar adverse events as when ziftomenib was used as a monotherapy in KOMET-001 [44,54]. Interestingly, compared to revumenib, there were minimal cases of QTc prolongation observed with ziftomenib. With the observed cases in patients taking medications with known side effects of QTc prolongation. The KOMET-017 study is currently underway to study the use of ziftomenib with ven/aza or 7+3 in newly diagnosed *NPM1*m and *KMT2A*r AML [43]. The current data on ziftomenib demonstrates promising outcomes in NPM1m and KMT2Ar AML with a familiar and controllable safety profile. The FDA recently approved ziftomenib as monotherapy for relapsed/refractory AML with NPM1 mutation on 13 November 2024 [55]. For the KMT2A indication, it is mostly in combination.

### 3.3 BN104

BN104 is currently being evaluated as a menin inhibitor in newly diagnosed (ND) and R/R AML [46]. The interim data from the Phase 1 study of BN104 as a monotherapy in R/R *KMT2A*r and *NPM1*m AML reports CR + CRh rates of 33.3% and ORR of 88.9% with 22.2% of patients progressing to transplant. Adverse events, including febrile neutropenia (15%), pneumonia (10%), grade 1 QTc prolongation (10%), and grade 2 differentiation syndrome (10%) were comparable to other menin inhibitors [46]. BN104 shows promise, but its clinical efficacy is yet to be determined until larger-scale studies are conducted, as only 20 patients have been treated so far.

### 3.4 Bleximenib

Bleximenib is under investigation in the setting as monotherapy for R/R disease, in combination with standard 7+3 intensive chemotherapy for ND disease, and in combination with ven/aza for R/R or ND AML. When bleximenib is used as a monotherapy for R/R *KMT2A*r or *NPM1*m AML, the CR + CRh rates and ORR, respectively, were 35% and 50% at 90/100 mg BID, 30% and 50% at 150 mg BID, and 23% and 39% at 45 mg BID. Notable safety data include only 1 patient (0.8%) with QTC prolongation and 14% of patients experiencing differentiation syndrome, with 7% at grade 3 or higher and 2 fatal events. The treatment-related adverse effects were consistent with those of other menin inhibitors, though the 150 mg BID dose was associated with a higher grade of thrombocytopenia and neutropenia [49]. Treatment with bleximenib with 7+3 chemotherapy in ND AML appears promising as ORR was 93% (*KMT2A*r: 83%, *NPM1*m: 100%) and CR + CRh rate was 86% (*KMT2A*r: 83%, *NPM1*m: 88%). Grade 3 or higher adverse events occurred in 95% of patients with febrile neutropenia (64%), thrombocytopenia (68%), anemia (41%), neutropenia (41%), and leukopenia (41%), remaining common. Interestingly, there were no reported cases of differentiation syndrome and only two episodes of grade 1 QTc prolongation observed, which were not due to bleximenib [48]. For patients with R/R or ND AML who are unfit for intensive chemotherapy, bleximenib was used with venetoclax and azacitidine. The ORR was 76% at 50 mg and 79% at 100 mg in the R/R subset and 77% at 50 mg and 92% at 100 mg bleximenib in the ND subset. The composite complete response rate (cCR) reported in the R/R group was 32% at 50 mg and 54% at 100 mg and the ND group was 62% at 50 mg and 85% at 100 mg. Differentiation syndrome occurred in 4% of patients, with the majority grade 2–3 and no events of QTc prolongation were observed [49]. Bleximenib represents a compelling therapeutic strategy for R/R AML or as an adjunct to standard chemotherapy in ND AML, especially in patients at risk for QTc prolongation due to existing medications or inherent cardiac risk factors.

### 3.5 Icovamenib (BMF-219)

Icovamenib is unique as the only covalent menin inhibitor that is being studied as monotherapy for R/R AML and also as a treatment for solid tumors and diabetes. Cohort 1 of the COVALENT-101 study is focused on the use of icovamenib in R/R AML and ALL. Five patients in the study were efficacy evaluable, with 1 patient achieving CR and 1 patient achieving CRi. The only grade 3 or higher adverse event was differentiation syndrome (13%), with no QTc prolongation in the 23 patients of the safety population [50]. Ongoing patient enrollment is necessary in reporting safety and efficacy data, as icovamenib is in the preliminary stages of clinical investigation.

### 3.6 Enzomenib

Enzomenib is presently being assessed as a singular therapeutic agent in the treatment of R/R AML. The recent analysis of the phase 1/2 study demonstrated CR + CRh rate of 37.5% and ORR of 62.5% in patients with R/R *KMT2A*r and *NPM1*m AML. Safety profile on enzomenib did not significantly differ from the other menin inhibitors, with reported cases of differentiation syndrome at 10.7% and QTc prolongation at 5% within the study population [51]. Enzomenib has demonstrated encouraging clinical activity in R/R AML, however it would be interesting to explore its application in ND *KMT2A*r or *NPM1*m AML.

## 4 Challenges and Potential Targeted Therapies

### 4.1 Acquired Resistance to Menin Inhibitors

Though past clinical trials demonstrate the promising activity of menin inhibitors in *KMT2A*r and *NPM1*m AML, the development of acquired resistance poses a significant challenge in their use as a single agent.

#### 4.1.1 MEN1 Mutations Confer Resistance to Menin Inhibitors

Molecular analyses from the AUGMENT-101 phase 1 study have shown that somatic mutations in *MEN1* can disrupt menin-inhibitor binding. Of note, patients developed resistance to revumenib as early as 2 cycles of treatment [56]. Common *MEN1* mutations that conferred resistance in both patients and xenografted models involved residues M327, G331, T349, and S160 [56,57]. Due to their proximity to the menin-inhibitor binding site, alterations to these residues introduced steric hindrance that diminished the binding affinity of menin inhibitors [56]. Somatic mutations in *MEN1* comprised approximately 40% of menin inhibitor-resistant AML cases in the study, thus other non-genetic pathways that contribute to menin inhibitor resistance have been explored.

#### 4.1.2 Epigenetic Modifications at Noncanonical Menin Targets Contribute to Menin Inhibitor Escape

In a recent study by Zhou et al., an epigenetic regulator within the Polycomb group protein family, Polycomb repressive complex 1.1 (PRC 1.1), was found to be involved in modulating resistance to menin inhibitors in *KMT2A*r leukemogenic cells [58]. Normally, PRC1.1 represses target genes by monoubiquinating histone H2A at lysine 119 (H2AK119ub) [59,60]. The repressive H2AK119ub signals were proposed to work with activating menin signals to regulate expression of various genes, such as *MYC* and *RUNX3*. Loss of PRC1.1 in *KMT2A*r AML models was found to epigenetically sustain or restore chromatin accessibility at menin’s noncanonical target loci, including *MYC*, thus enabling their continued transcription even under menin inhibition. The resulting overexpression of *MYC* is associated with reduced myeloid or monocytic differentiation, conferring resistance to menin inhibitors through a menin-independent mechanism [58]. Combining MYC inhibition with menin inhibitors could offer a novel and potentially synergistic approach that also helps reduce resistance to menin-targeted therapy.

#### 4.1.3 Co-Mutations May Reduce the Overall Efficacy of Menin Inhibitors

Mutations at other gene loci often co-occur with *KMT2A* rearrangements in AML. Genes involved in cellular signaling, particularly *NRAS*, *KRAS*, and *FLT3*-TKD were most frequently detected in patients with *KMT2A*r AML, followed by the protooncogene *TP53* and chromatin-modifying genes, tet methylcytosine dioxygenase 2 (*TET2*) and DNA methyltransferase 3 alpha (*DNMT3A*) [8,61,62]. These co-mutations have been linked to worse prognosis with lower median OS and response rates to conventional therapies in adult AML patients with *KMT2A*r. Pediatric populations share some of the co-mutations that occur in adults, particularly the RAS pathway genes, but also present with a rather high incidence of SET domain containing 2, histone lysine methyltransferase (*SETD2*) mutations [63,64]. Both were correlated with worse 5-year OS. Though many of these mutations have not yet been shown to be direct drivers of menin inhibitor resistance, they may increase the risk of therapeutic failure and reduce the overall efficacy of menin inhibitors. Thus, combination therapies and early intervention with menin inhibitors may be warranted to prevent adverse outcomes from co-mutations.

### 4.2 Therapies to Overcome Acquired Resistance to Menin Inhibitors

Combination therapies incorporating BCL2, FLT3, IDH1/IDH2, or enhancer of zeste homolog 2 (EZH2) inhibitors may enhance the activity of menin inhibitors and provide complementary mechanisms to overcome resistance. The BCL2 inhibitor, venetoclax, and FLT3 inhibitor, gilteritinib, are currently being tested in clinical trials, showing improved ORR and CR rates than a single agent alone (Table 2).

#### 4.2.1 BCL2 Inhibitors

Aberrant expression of *HOX*/*MEIS1* genes in AML leads to upregulation of their downstream targets, such as the anti-apoptotic gene, *BCL2*. Preclinical studies have shown that menin inhibition can sensitize *KMT2A*r AML cells to venetoclax by downregulating expression of *HOX/MEIS1*-driven transcriptional programs [65]. Simultaneously, inhibition of the residual BCL2 by venetoclax lowers the cells’ apoptotic threshold and exerts a cytotoxic effect. This synergistic activity has the potential to overcome menin inhibitor resistance driven by epigenetic escape, as seen in PRC1.1 loss. Zhou et al. demonstrated that PRC1.1-depleted AML cells exhibit diminished monocytic differentiation gene signatures and adopt a primitive state, which was associated with increased venetoclax sensitivity [58]. Mechanistically, this is consistent with the normal upregulation of BCL2 in early myeloid progenitors and its downregulation during monocytic differentiation, indicating that the primitive state of PRC1.1-deficient cells may underlie their sensitivity to BCL2 inhibition [65,66]. Thus, the combination of BCL2 and menin inhibitors may not only enhance each other’s antileukemic activities but also overcome the possibility of menin inhibitor resistance.

#### 4.2.2 FLT3 Inhibitors

In addition to *BCL2*, the receptor tyrosine kinase (RTK) gene, *FLT3*, is an upregulated downstream target of the *HOX/MEIS1* transcriptional program. Menin inhibitors can downregulate *FLT3* expression, but in *FLT3*-mutated AML, the RTK is constitutively activated, leading to activation of pro-leukemogenic signaling pathways, Janus kinase/signal transducer and activator of transcription (JAK/STAT), Ras-mitogen-activated protein kinase (RAS/MAPK), and phosphatidylinositol 3-kinase/protein kinase B/mammalian target of rapamycin (PI3K/AKT/mTOR) [67–69]. Gilteritinib is a type I FLT3-inhibitor that operates by blocking the receptor’s ATP binding site and preventing its autophosphorylation [68,70]. While menin inhibitors reduce *FLT3* expression at the transcriptional level, FLT3 inhibitors further drive down residual kinase activity. This synergistic combination is especially useful in *KMT2A*r or *NPM1*m AML patients with FLT3 co-mutations. Currently, gliteritinib and revumenib are in phase 1 of clinical trials for patients with R/R *FLT3*-mutated AML with *KMT2A*r or *NPM1*m (Table 1).

#### 4.2.3 IDH1/IDH2 Inhibitors

*IDH1*/*IDH2* mutations occur less commonly with *KMT2A*r AML but are associated with about 25% of *NPM1*m AML cases [71]. Though IDH1/IDH2 inhibitors are not yet in clinical trials with menin inhibitors, preclinical studies suggest enhanced therapeutic use when combined. Mutated IDH1/IDH2 enzymes convert alpha-ketoglutarate to D-2-hydroxyglutarate, which inhibits activity of DNA and histone methylases important for epigenetic regulation of cellular differentiation [72]. Combined use of IDH1/IDH2 and menin inhibitors further promotes differentiation of leukemic cells at the epigenetic and genetic levels, respectively and has shown a more significant reduction of *HOX*/*MEIS1* expression than seen with single agents [71].

#### 4.2.4 EZH2 Inhibitors

Recent findings have demonstrated that EZH2 inhibition synergizes with menin inhibition [73]. When menin is inhibited, KMT2A/B is redistributed to bivalent promoters, increasing H3K4me3 and displacing PRC2, thereby initiating transcriptional de-repression. Concurrent inhibition of EZH2, which is the catalytic subunit of PRC2, prevents re-methylation of H3K27, amplifying the de-repression and promoting activation of silenced gene programs, including MHC-1 antigen presentation pathways. Preclinical models have shown that this combination leads to enhanced immune recognition and cytotoxic T-cell-mediated tumor clearance. The rationale for combination epigenetic therapy using menin and EZH2 inhibitors to overcome resistance and restore differentiation and immune responsiveness in AML remains an area of study.

#### 4.2.5 Other Menin Inhibitors

Newer menin inhibitors are currently under development that aim to reduce adverse outcomes and combat acquired resistance that were seen in revumenib clinical trials. In the KOMET-001 phase 1 study of ziftomenib, 1 of 29 patients (~3.4%) developed a resistance mutation compared to 12 of 31 patients (~39%) in the AUGMENT-101 trial of revumenib, though this may be due to different sensitivities of their assays. However, further *in vitro* studies have shown that ziftomenib was able to maintain its activity against G331 and T349 mutated menin [42]. In addition, a second-generation menin inhibitor, BTC-86, was described to overcome the steric clash introduced by all *MEN1* acquired mutations by adopting a unique binding configuration, though studies are still underway [74].

## 5 Biomarkers for Monitoring Response and Resistance in Menin Inhibitor Therapy

### 5.1 Biomarkers to Track Clones and Response to Disease

Identifying and monitoring molecular biomarkers are essential to optimize the therapeutic use of menin inhibitors in AML, particularly in genetically defined subgroups such as *KMT2A*r and *NPM1*m AML. One of the most promising biomarkers of treatment response is the expression of *HOX* (A and/or B) genes and their transcriptional cofactor *MEIS1*, both aberrantly upregulated in these disease subsets. These genes are directly regulated by menin and are consistently downregulated in response to effective menin inhibition, providing a dynamic measure of therapeutic efficacy. Their expression levels can be tracked using RNA sequencing (RNA-Seq), which allows real-time assessment of transcriptional responses during therapy [35,75].

A critical biomarker of response and resistance is the emergence of *MEN1* gene mutations. These mutations can be acquired under therapeutic pressure and have been shown to mediate resistance to menin inhibitors by altering the drug-binding site or associated chromatin remodeling functions. *MEN1* mutations often precede morphologic or clinical relapse, making early detection through next-generation sequencing (NGS) essential to adjust treatment strategies accordingly [76,77]. In addition to *MEN1* alterations, the evolution of other AML-associated mutations may contribute to therapeutic resistance, highlighting the importance of comprehensive genomic surveillance over time [75].

### 5.2 Key Laboratory Methods for Biomarker Assessment (RNA-Seq, FISH, ddPCR, DNA Sequencing)

A multipronged diagnostic approach is necessary for effective monitoring of menin inhibitor therapy (Table 5).

**Table 5:** Molecular and functional biomarkers in menin inhibitor therapy

Biomarker	Method	Clinical/Translational application	Validation status
HOXA Cluster (HOXA9, HOXA10)	RNA-seq, qPCR	Dynamic marker of response, downregulation correlates with differentiation and clinical response	Validated—pharmacodynamic response marker in AUGMENT-101
HOXB Cluster	RNA-seq	Reflects menin-KMT2A transcriptional dependency and decreases with effective inhibition	Investigational
MEIS1	RNA-seq	Surrogate indicator of HOX pathway suppression and early molecular response	Validated
MEN1 (M327, G331, T349, S160)	PCR, NGS, ddPCR	Predicts and monitors on-therapy resistance; emerges prior to morphologic relapse	Validated
PRC1.1 complex (BMI1, PCGF1, RING1A/B)	ChIP-seq, ATAC-seq (research)	Loss indicates menin-independent chromatin accessibility and *MYC*-mediated resistance	Preclinical
MYC	RNA-seq	Overexpression after menin inhibition signals epigenetic escape; rationale for MYC co-targeting	Investigational
*KMT2A* rearrangement (*KMT2A*r)	FISH, RT-PCR, NGS	Diagnostic; identifies canonical menin-dependent AML; monitors persistence post-therapy	*Validated*
*NPM1* mutation	PCR, NGS, ddPCR	Diagnostic; tracks MRD; predicts response to menin inhibition	*Validated*
*NUP98* rearrangement	FISH, RNA-seq	Expanding indication; predicts potential menin-sensitive subset	*Emerging evidence*
*TP53* mutation	NGS	Associated with attenuated venetoclax synergy and possibly reduced menin inhibitor efficacy	*Exploratory*
*RAS* pathway mutations (*NRAS*, *KRAS*, *FLT3*-TKD)	NGS	May drive primary resistance or relapse through alternative signaling	*Investigational*
*XPO1*-mediated *NPM1* localization	Immunofluorescence, subcellular fractionation	Correlates with sustained HOX/MEIS activation and menin dependence	*Preclinical*
MRD transcript detection (*KMT2A*-fusion, *NPM1*, *HOXA9*)	ddPCR, qPCR, flow cytometry	Tracks residual disease and early relapse; integrates with treatment decision algorithms	*Validated* (ELN-aligned)
Circulating tumor DNA (ctDNA) for *MEN1*/*KMT2A*	NGS-based ctDNA panels	Non-invasive monitoring of clonal evolution and emerging resistance	*Investigational*

Note: RNA-seq, RNA sequencing; qPCR, quantitative polymerase chain reaction; NGS, next generation sequencing; ddPCR, droplet digital polymerase chain reaction; ChIp-seq, chromatin immunoprecipitation sequencing; ATAC-seq, assay for transposase-accessible chromatin with sequencing; FISH, fluorescence *in situ* hybridization; RT-PCR, reverse transcription-polymerase chain reaction; PCR, polymerase chain reaction; ctDNA, circulating tumor DNA; AML, acute myeloid leukemia; MRD, minimal residual disease.

RNA-Seq is indispensable for quantifying *HOX* and *MEIS1* gene expression and identifying transcriptional changes associated with response or resistance. DNA sequencing, particularly through NGS panels, enables the detection of *KMT2A*r, *NPM1*m, *MEN1* mutations, and clonal evolution. Fluorescence *in situ* hybridization (FISH) is a complementary tool for confirming *KMT2A* rearrangements at the chromosomal level [78]. MRD assessment via sensitive methods such as flow cytometry, droplet digital PCR (ddPCR), or quantitative PCR also provides valuable insight into treatment depth and relapse risk [35,78]. For MRD detection and early relapse prediction, ddPCR offers high sensitivity to capture low-frequency resistant clones or residual leukemic burden, particularly in settings where other tests may lack sufficient resolution [79]. As menin inhibitors are adopted into clinical practice, it is imperative to continue clinical and translational research to refine and optimize the use of menin inhibitors further. Biomarkers need their prognostic and predictive values validated, which can then be applied to further develop interventions and circumvent emerging resistance.

## 6 Future Directions

Menin inhibitors have demonstrated promising therapeutic activity in patients with R/R *KMT2A*r AML and have since expanded their use to include those with *NPM1* mutations and *NUP98* rearrangements. Clinical trials have enrolled a substantial proportion of older adults, many of whom were unfit for intensive chemotherapy, and have reported meaningful ORR with manageable toxicity profiles. The efficacy and safety profiles of menin inhibitors in pediatric patients appear comparable to those observed in adults. However, pediatric cohorts have constituted only a small subset of the study populations. Thus, the efficacy and long-term effects of menin inhibitors in children remain to be fully elucidated. Given the mechanism of action and adverse events observed in clinical studies, potential long-term risks may include cardiotoxicity and sustained cytopenias. Therefore, ongoing surveillance and long-term follow-up of menin inhibitor-treated pediatric patients are warranted.

Thus far, acute adverse events from menin inhibitor use seem to have been manageable. One of the growing concerns of menin inhibitors, though, is acquired resistance to menin inhibitors. Recent data from Bourgeois et al. have provided key insights into the impact of *MEN1* mutations on the efficacy of menin inhibitors [80]. Specific *MEN1* mutations have been shown to mediate resistance, with important implications for patient selection and therapeutic sequencing. The Met327 mutation in MEN1 leads to class-wide resistance across all menin inhibitors studied, significantly impairing drug binding. Other mutations, including Cys334, Glu368, and Val372, show selective resistance to individual compounds, suggesting drug-specific vulnerabilities. Proliferation assays in *MEN1*-mutated MOLM13 cells revealed 10x shifts in GI50 for JNJ and Sumitomo agents, and 30×–75× shifts for KO-539, DSP-5336, and SNDX-5613 [80]. These findings suggest that *MEN1* mutational profiling may be warranted in patients receiving menin inhibitors, especially in relapsed/refractory settings. Ongoing drug development should aim to address class resistance and develop next-generation agents with efficacy in MEN1-mutant contexts.

Combination therapies with BCL2, FLT3, and IDH1/IDH2 inhibitors have been proposed to combat acquired resistance to menin inhibitors. However, their combined use may introduce additional complications inherent to each agent, particularly in increased QTc prolongation and differentiation syndrome. Given the heterogeneous nature of leukemic cell populations, drugs like venetolax can impose a selection pressure and allow cells that can bypass the drug’s main mechanism of action to survive and proliferate [66]. Previous studies have shown that AML patients with *TP53* mutations have poor response to venetoclax, which can even reduce its synergistic effects with revumenib [81,82]. Rather, a myeloid cell leukemia sequence 1 (MCL1) inhibitor was proposed to work more effectively with revumenib in *TP53* mutant *KMT2A*r cells [81]. Currently, there are no clinical trials reporting the efficacy of menin inhibitors in *KMT2A*r AML patients with concurrent *TP53* mutations. Though some trials have included patients with *TP53* mutations, efficacy results for this subgroup have not been separately reported. *TP53* mutations are known to confer resistance to many targeted therapies, thus menin inhibitor efficacy and the combination therapies to use with this co-mutation should be explored further.

In addition, the combined use of menin inhibitors with FLT3 or IDH1/IDH2 inhibitors have the potential to exacerbate the acute toxicities seen with menin inhibitor monotherapy in clinical trials, particularly QTc prolongation and differentiation syndrome. Since even the mildest presentation of differentiation syndrome can progress quickly, it is up to providers to monitor patients carefully and adjust dosages as needed, especially when using combined therapies. To further assess the efficacy of combination therapy vs. monotherapy, a comparison of median OS would be useful. While monotherapy with menin inhibitors shows a median OS of ~6–8 months (Table 6), the combination therapy mOS is still unknown, given the early stages of their clinical trials. As menin inhibitor use is studied in triple therapy combinations with HMA/Ven, there is also interest to explore quadruple therapy in *FLT3-*mutated or *IDH1/2*-mutated patients. Though there may be a significant improvement in efficacy, toxicities will need to be closely monitored.

**Table 6:** Comparative analysis of menin inhibitors in clinical trials

Agent	Phase	ORR (%)	CR/CRh (%)	mDoR (Months)	mOS (Months)	Patients to alloHCT
Ziftomenib [42,43]	Phase 1	17%	11%	3.1	5.4	0
Bleximenib [47–49]	Phase 1	46%	21%	6.5	6.0	7 (1 responder)
Enzomenib [51]	Phase 1	59%	23%	NR	NR	NR
Revumenib [37–39,53]	Phase 2	64%	23%	6.4	8.0	39 (responders)

Note: Summary of efficacy outcomes from early-phase clinical trials of key menin inhibitors: ziftomenib, bleximenib, enzomenib, and revumenib targeting relapsed/refractory AML with *KMT2A* rearrangement and/or *NPM1* mutation. NR, Not Reported; mDoR, Median Duration of Response; mOS, Median Overall Survival.

The use of menin inhibition in the maintenance setting is also being explored, both for post-induction maintenance and post-transplant maintenance. Given the oral route of administration and relatively well-tolerated safety profile, continued single-agent maintenance may prove to be an effective method to reduce relapse rates, however there is currently no long-term safety or efficacy data. AML with *KMT2A*r or *NPM1*m has substantial relapse risk even after transplant. Early evidence supports maintenance therapy post-transplant. In an MSKCC study, 9 patients received revumenib after allogeneic SCT (for 23 to 588 days) as maintenance [83]. CRc was maintained in 6 of 9 patients after HSCT and maintenance revumenib. One patient with reported MRD after HSCT converted to MRD-negative status following initiation of revumenib maintenance therapy. Overall, MRD-negative remissions were maintained in 5 patients as of the data cutoff.Besides the next generation of menin inhibitors, menin degraders are also being researched. Efforts are exploring proteolysis-targeting chimeras that lead to menin degradation rather than inhibition. A menin degrader could eliminate menin protein entirely, potentially overcoming high menin levels or mutations that affect only the binding site. Preclinical degraders against menin have shown potent cell killing in MLL-r models, but none are in clinical trials yet. Degraders also introduce novel risks, such as off-target ubiquitination.

## 7 Conclusion

The development of menin inhibitors has introduced a new therapeutic opportunity for patients with *KMT2A*r and *NPM1*m AML. These subtypes were once considered biologically adverse but are now targetable through inhibition of the menin–KMT2A interaction. Multiple agents, notably revumenib and ziftomenib, have demonstrated significant activity in relapsed or refractory AML and are advancing toward frontline use in combination regimens. Early results consistently show high response rates and the potential to bridge patients to curative therapies such as allogeneic transplantation.

However, resistance mechanisms, especially acquired *MEN1* mutations and epigenetic reprogramming, have already been discovered and are under further study. Emerging data suggest that combinations with agents such as venetoclax, hypomethylating agents, IDH1/IDH2 or FLT3 inhibitors, and even EZH2 inhibitors may mitigate resistance and extend therapeutic benefit. Biomarker-informed strategies, including serial monitoring of HOX/MEIS1 expression and clonal evolution through NGS and ddPCR, are essential for optimizing treatment and anticipating relapse. As the field continues to mature, menin inhibition is poised to become a strategy in the molecularly targeted management of AML and its use in combination or maintenance has the potential to provide significant clinical benefit.

## Data Availability

Not applicable.

## Appendix A

**Table A1:** Menin inhibitor clinical trials

Drug	Phase	NCT	Regimen	Diagnosis	Mutation	Population	Country/Region	Status
	Phase 1 (City of Hope)	NCT06575296	Monotherapy	AML, ALL (post allogeneic HSCT)	*KMT2A, NPM1*	≥2Y	USA	Recruiting
	Phase 1/2 (Syndax Pharmaceuticals)	NCT04065399	Monotherapy (+/− CYP3A4 inhibitor)	AML, ALL	*KMT2A, NPM1, NUP98*	≥1M	USA, Australia, Canada, France, Germany, Israel, Italy, Lithuania, Netherlands, Puerto Rico, Spain	Recruiting
	Phase 2 (M.D. Anderson)	NCT06229912	Monotherapy	AML, ALL	*KMT2A, NPM1, NUP98, NUP214* (upregulation of HOX genes)	≥12Y	USA	Recruiting
	Phase 1	NCT06222580	Combination: Revumenib + Gilteritinib	AML	*KMT2A, NPM1, FLT3*	≥18Y	USA	Recruiting
	Phase 1	NCT06313437	Combination: Revumenib + Cytarabine/Daunorubicin + Midostaurin	AML	*NPM1, FLT3*	≥18Y and ≤75Y	USA	Recruiting
	Phase 1 (National Cancer Institute)	NCT05886049	Combination: Revumenib + Daunorubicin/Cytarabine	AML	*KMT2A, NPM1*	≥18Y and ≤75Y	USA	Recruiting
Revumenib (SNDX-5613)	Phase 1	NCT06177067	Combination: Revumenib +Azacitidine/ Venetoclax	AML, ALAL	*KMT2A, NPM1, NUP98, NUP214*	≥1Y and ≤30Y	USA	Recruiting
	Phase 1	NCT07052994	Combination: Revumenib + Daunorubicin/ Cytarabine	AML, MPAL	*KMT2, NPM1, NUP98, UBTF-ITD*	≥6M and ≤21Y	USA	Not Yet Recruiting
	Phase 1	NCT06226571	Combination: Revumenib + Cytarabine + Daunorubicin OR Idarubicin	AML	*KMT2, NPM1, NUP98*	≥18Y and ≤75Y	USA, Australia, Spain, UK	Recruiting
	Phase 1/2 (M.D. Anderson)	NCT05360160	Combination: Revumenib + Decitabine/Cedazuridine (ASTX727) + Venetoclax	AML	*KMT2A, NPM1, NUP98*	≥12Y	USA	Recruiting
	Phase 1/2 (M.D. Anderson)	NCT06284486	Combination: Revumenib + Venetoclax	AML	*KMT2A, NPM1, NUP98*	≥12Y	USA	Recruiting
	Phase 2 (Children’s Oncology Group)	NCT05761171	Combination: Revumenib + Fludarabine/Cytarabine	ALL	*KMT2A*	≥1M and <6Y	USA, Canada	Recruiting
	Phase 3	NCT06652438	Combination: Revumenib + Azacitidine/Venetoclax	AML	*KMT2A, NPM1*	≥18Y	Germany, Netherlands, UK	Recruiting
	Phase 3	NCT07211958	Combination: Intensive Chemotherapy + Revumenib OR Placebo	AML	*NPM1*	≥12Y	N/A	Not Yet Recruiting
	KO-MEN-001 Phase 1/2	NCT04067336	Monotherapy (+/− midazolam or itraconazole)	AML	*KMT2A, NPM1*	≥18Y	USA, Belgium, Canada, France, Germany, Italy, Poland, Spain, UK	Recruiting
	Phase 2	NCT06930352	Monotherapy	AML (not eligible for standard therapy)	*KMT2A, NPM1*	≥18Y	USA	Not Yet Recruiting
	KO-MEN-007 Phase 1	NCT05735184	Combination: Ziftomenib + Venetoclax/Azacitidine OR Venetoclax OR Cytarabine/Daunorubicin	AML	*KMT2A, NPM1*	≥18Y	USA	Recruiting
Ziftomenib	Phase 1 (M.D. Anderson)	NCT06448013	Combination: Ziftomenib + Venetoclax/Gemtuzumab	AML, MPAL	*KMT2A, NPM1, NUP98, UBTF-ITD*	≥3Y and ≤21Y	USA	Recruiting
	Phase 1	NCT06376162	Combination: Ziftomenib + Cytarabine/Fludarabine	AML	*KMT2A, NPM1, NUP98*	≤21Y	USA, Austria, Canada, France, Netherlands, Spain	Recruiting
	Phase 1	NCT06397027	Combination: Ziftomenib + Venetoclax/Azacitidine	AML, MPAL	*KMT2A, NPM1, NUP98*	≥2Y and ≤21Y	USA	Recruiting
	Phase 1	NCT06769490	Combination: Ziftomenib + Quizartinib	AML, MPAL	*KMT2A, NPM1, NUP98*	≥18Y	USA	Recruiting
	Phase 1	NCT06001788	Combination: Ziftomenib + Fludarabine/Idarubicin OR Cytarabine OR Gliteritinib	AML	*KMT2A, NPM1, FLT3*	≥18Y	USA, Italy, Spain	Recruiting
	Phase 3	NCT07007312	Combination: Ziftomenib + Venetoclax/Azacitidine OR Cytarabine/Daunorubicin	AML	*KMT2A, NPM1, FLT3*	≥18Y	Not provided	Not Yet Recruiting
BN104	Phase 1/2 (BioNova Pharmaceuticals)	NCT06052813	Monotherapy	AML, ALL	*KMT2A, NPM1*	≥18Y	China	Recruiting
	Phase 1/2	NCT06746519	Combination: BN104 + Venetoclax/Azacitidine	AML	*KMT2A, NPM1, NUP98*	≥18Y	China	Recruiting
	Phase 1/2 (Janssen R&D)	NCT04811560	Monotherapy	AML, ALL	Phase 1: *KMT2A, NPM1, NUP98, NUP214*; Phase 2: *KMT2A, NPM1*	Phase 1: ≥12Y and <18Y; Phase 2: ≥18Y	USA, Australia, Brazil, Canada, China, France, Israel, Japan, Korea, Spain, Taiwan, UK	Recruiting
Bleximenib (JNJ-75276617)	Phase 1 (Janssen R&D)	NCT05453903	Combination: Bleximenib + Venetoclax/Azacitidine OR Cytarabine/Daunorubicin OR Idarubicin	AML	*KMT2A, NPM1, NUP98, NUP214*	≥12Y	US, Australia, Canada, France, Germany, Italy, Spain, UK	Recruiting
	Phase 3	NCT06852222	Combination: Azacitidine/Venetoclax + Bleximenib OR Placebo	AML	*KMT2A, NPM1*	≥18Y	USA, Australia, Belgium, Brazil, Canada, China, Czechia, Denmark, France, Germany, Greece, Israel, Italy, Japan, Poland, Portugal, South Korea, Spain, Taiwan, Turkey, UK	Recruiting
BMF-219	COVALENT-101 Phase 1 (Biomea Fusion)	NCT05153330	Monotherapy (+/− CYP3A4 inhibitor)	AML, ALL, DLBCL, MM, CLL/SLL	*KMT2A/MLL1, NPM1*	≥18Y	USA, Greece, Italy, Netherlands, Spain	Active, Not Recruiting
Enzomenib (DSP-5336)	Phase 1/2 (Sumitomo Pharma America)	NCT04988555	Monotherapy (+/− CYP3A4 inhibitor); Combination: Enzomenib + Venetoclax/Azacitidine OR Gilteritinib	AML, ALL, MM, MDS	*MLL1, NPM1*	≥18Y	USA, Belgium, Canada, France, Italy, Japan, Korea, Singapore, Spain, Taiwan, UK	Recruiting

Note: All up-to-date clinical trial information can be found on clinicaltrials.gov. AML, acute myeloid leukemia; ALL, acute lymphoblastic leukemia; HSCT, hematopoietic stem cell transplantation; ALAL, acute leukemia of ambiguous lineage; MPAL, mixed phenotype acute leukemia; DLBCL, diffuse large B-cell lymphoma; MM, multiple myeloma; CLL, chronic lymphocytic leukemia; SLL, small lymphocytic lymphoma; MDS, myelodysplastic syndrome; USA, United States of America; UK, United Kingdom.

## References

[1] DiNardo CD, Erba HP, Freeman SD, Wei AH. Acute myeloid leukaemia. Lancet. 2023;401(10393):2073–86. doi:10.1016/S0140-6736(23)00108-3; 37068505

[2] Khwaja A, Bjorkholm M, Gale RE, Levine RL, Jordan CT, Ehninger G, et al. Acute myeloid leukaemia. Nat Rev Dis Primers. 2016;2:16010. doi:10.1038/nrdp.2016.10; 27159408

[3] Shimony S, Stahl M, Stone RM. Acute myeloid leukemia: 2023 update on diagnosis, risk-stratification, and management. Am J Hematol. 2023;98(3):502–26. doi:10.1002/ajh.26822; 36594187

[4] Papaemmanuil E, Gerstung M, Bullinger L, Gaidzik VI, Paschka P, Roberts ND, et al. Genomic classification and prognosis in acute myeloid leukemia. N Engl J Med. 2016;374(23):2209–21. doi:10.1056/nejmoa1516192; 27276561 PMC4979995

[5] Bullinger L, Döhner K, Döhner H. Genomics of acute myeloid leukemia diagnosis and pathways. J Clin Oncol. 2017;35(9):934–46. doi:10.1200/JCO.2016.71.2208; 28297624

[6] Issa GC, Zarka J, Sasaki K, Qiao W, Pak D, Ning J, et al. Predictors of outcomes in adults with acute myeloid leukemia and KMT2A rearrangements. Blood Cancer J. 2021;11(9):162. doi:10.1038/s41408-021-00557-6; 34588432 PMC8481264

[7] Dali SA, Al-Mashdali AF, Kalfah A, Mohamed SF. Menin inhibitors in KMT2A-rearranged and NPM1-mutated acute leukemia: a scoping review of safety and efficacy. Crit Rev Oncol Hematol. 2025;213:104783. doi:10.1016/j.critrevonc.2025.104783; 40441466

[8] Bataller A, Goulart HE, Issa GC, DiNardo CD, Daver N, Kadia T, et al. Characteristics and outcomes of newly diagnosed acute myeloid Leukemia with KMT2A rearrangements. Leukemia. 2025;39(7):1640–9. doi:10.1038/s41375-025-02634-2; 40346311

[9] Yokoyama A, Wang Z, Wysocka J, Sanyal M, Aufiero DJ, Kitabayashi I, et al. Leukemia proto-oncoprotein MLL forms a SET1-like histone methyltransferase complex with menin to regulate *Hox* gene expression. Mol Cell Biol. 2004;24(13):5639–49. doi:10.1128/MCB.24.13.5639-5649.2004; 15199122 PMC480881

[10] Yokoyama A, Somervaille TCP, Smith KS, Rozenblatt-Rosen O, Meyerson M, Cleary ML. The menin tumor suppressor protein is an essential oncogenic cofactor for MLL-associated leukemogenesis. Cell. 2005;123(2):207–18. doi:10.1016/j.cell.2005.09.025; 16239140

[11] Maillard I, Chen YX, Friedman A, Yang Y, Tubbs AT, Shestova O, et al. Menin regulates the function of hematopoietic stem cells and lymphoid progenitors. Blood. 2009;113(8):1661–9. doi:10.1182/blood-2009-01-135012; 19228930 PMC2647667

[12] Grembecka J, Belcher AM, Hartley T, Cierpicki T. Molecular basis of the mixed lineage leukemia-menin interaction: implications for targeting mixed lineage leukemias. J Biol Chem. 2010;285(52):40690–8. doi:10.1074/jbc.M110.172783; 20961854 PMC3003368

[13] McMahon KA, Hiew SY, Hadjur S, Veiga-Fernandes H, Menzel U, Price AJ, et al. Mll has a critical role in fetal and adult hematopoietic stem cell self-renewal. Cell Stem Cell. 2007;1(3):338–45. doi:10.1016/j.stem.2007.07.002; 18371367

[14] Li X, Song Y. Structure, function and inhibition of critical protein-protein interactions involving mixed lineage leukemia 1 and its fusion oncoproteins. J Hematol Oncol. 2021;14(1):56. doi:10.1186/s13045-021-01057-7; 33823889 PMC8022399

[15] Issa GC, Ravandi F, DiNardo CD, Jabbour E, Kantarjian HM, Andreeff M. Therapeutic implications of menin inhibition in acute leukemias. Leukemia. 2021;35(9):2482–95. doi:10.1038/s41375-021-01309-y; 34131281

[16] Ernst P, Kyei SP, Yokoyama A. KMT2A-rearranged leukemia: from mechanism to drug development. Exp Hematol. 2025;151:105247. doi:10.1016/j.exphem.2025.105247; 40921261 PMC12626164

[17] Meyer C, Larghero P, Almeida Lopes B, Burmeister T, Gröger D, Sutton R, et al. The KMT2A recombinome of acute leukemias in 2023. Leukemia. 2023;37(5):988–1005. doi:10.1038/s41375-023-01877-1; 37019990 PMC10169636

[18] Janssens DH, Meers MP, Wu SJ, Babaeva E, Meshinchi S, Sarthy JF, et al. Automated CUT&Tag profiling of chromatin heterogeneity in mixed-lineage leukemia. Nat Genet. 2021;53(11):1586–96. doi:10.1038/s41588-021-00941-9; 34663924 PMC8571097

[19] Krivtsov AV, Armstrong SA. MLL translocations, histone modifications and leukaemia stem-cell development. Nat Rev Cancer. 2007;7(11):823–33. doi:10.1038/nrc2253; 17957188

[20] Caslini C, Yang Z, El-Osta M, Milne TA, Slany RK, Hess JL. Interaction of MLL amino terminal sequences with menin is required for transformation. Cancer Res. 2007;67(15):7275–83. doi:10.1158/0008-5472.CAN-06-2369; 17671196 PMC7566887

[21] Chen YX, Yan J, Keeshan K, Tubbs AT, Wang H, Silva A, et al. The tumor suppressor menin regulates hematopoiesis and myeloid transformation by influencing Hox gene expression. Proc Natl Acad Sci U S A. 2006;103(4):1018–23. doi:10.1073/pnas.0510347103; 16415155 PMC1326489

[22] Issa GC, Aldoss I, DiPersio J, Cuglievan B, Stone R, Arellano M, et al. The menin inhibitor revumenib in KMT2A-rearranged or NPM1-mutant leukaemia. Nature. 2023;615(7954):920–4. doi:10.1038/s41586-023-05812-3; 36922593 PMC10060155

[23] Struski S, Lagarde S, Bories P, Puiseux C, Prade N, Cuccuini W, et al. NUP98 is rearranged in 3.8% of pediatric AML forming a clinical and molecular homogenous group with a poor prognosis. Leukemia. 2017;31(3):565–72. doi:10.1038/leu.2016.267; 27694926

[24] Bertrums EJM, Smith JL, Harmon L, Ries RE, Wang YJ, Alonzo TA, et al. Comprehensive molecular and clinical characterization of NUP98 fusions in pediatric acute myeloid leukemia. Haematologica. 2023;108(8):2044–58. doi:10.3324/haematol.2022.281653; 36815378 PMC10388277

[25] Kim N, Choi YJ, Cho H, Jang JE, Lee ST, Song J, et al. NUP98 is rearranged in 5.0% of adult East Asian patients with AML. Blood Adv. 2024;8(19):5122–5. doi:10.1182/bloodadvances.2024012960; 39158088 PMC11460442

[26] Oka M, Mura S, Otani M, Miyamoto Y, Nogami J, Maehara K, et al. Chromatin-bound CRM1 recruits SET-Nup214 and NPM1c onto HOX clusters causing aberrant HOX expression in leukemia cells. elife. 2019;8:e46667. doi:10.7554/eLife.46667; 31755865 PMC6874418

[27] Falini B, Sorcini D, Perriello VM, Sportoletti P. Functions of the native NPM1 protein and its leukemic mutant. Leukemia. 2025;39(2):276–90. doi:10.1038/s41375-024-02476-4; 39690184

[28] Leong SM, Tan BX, Bte Ahmad B, Yan T, Chee LY, Ang ST, et al. Mutant nucleophosmin deregulates cell death and myeloid differentiation through excessive caspase-6 and-8 inhibition. Blood. 2010;116(17):3286–96. doi:10.1182/blood-2009-12-256149; 20606168

[29] Brunetti L, Gundry MC, Sorcini D, Guzman AG, Huang YH, Ramabadran R, et al. Mutant NPM1 maintains the leukemic state through HOX expression. Cancer Cell. 2018;34(3):499–512.e9. doi:10.1016/j.ccell.2018.08.005; 30205049 PMC6159911

[30] Uckelmann HJ, Haarer EL, Takeda R, Wong EM, Hatton C, Marinaccio C, et al. Mutant NPM1 directly regulates oncogenic transcription in acute myeloid leukemia. Cancer Discov. 2023;13(3):746–65. doi:10.1158/2159-8290.CD-22-0366; 36455613 PMC10084473

[31] Xu H, Valerio DG, Eisold ME, Sinha A, Koche RP, Hu W, et al. NUP98 fusion proteins interact with the NSL and MLL1 complexes to drive leukemogenesis. Cancer Cell. 2016;30(6):863–78. doi:10.1016/j.ccell.2016.10.019; 27889185 PMC5501282

[32] Fisher JN, Thanasopoulou A, Juge S, Tzankov A, Bagger FO, Mendez MA, et al. Transforming activities of the NUP98-KMT2A fusion gene associated with myelodysplasia and acute myeloid leukemia. Haematologica. 2020;105(7):1857–67. doi:10.3324/haematol.2019.219188; 31558671 PMC7327646

[33] Heikamp EB, Henrich JA, Perner F, Wong EM, Hatton C, Wen Y, et al. The menin-MLL1 interaction is a molecular dependency in NUP98-rearranged AML. Blood. 2022;139(6):894–906. doi:10.1182/blood.2021012806; 34582559 PMC8832476

[34] Kwon MC, Thuring JW, Querolle O, Dai X, Verhulst T, Pande V, et al. Preclinical efficacy of the potent, selective menin-KMT2A inhibitor JNJ-75276617 (bleximenib) in KMT2A- and NPM1-altered leukemias. Blood. 2024;144(11):1206–20. doi:10.1182/blood.2023022480; 38905635 PMC11419783

[35] Candoni A, Coppola G. A 2024 update on menin inhibitors. A new class of target agents against KMT2A-rearranged and NPM1-mutated acute myeloid leukemia. Hematol Rep. 2024;16(2):244–54. doi:10.3390/hematolrep16020024; 38651453 PMC11036224

[36] Borkin D, He S, Miao H, Kempinska K, Pollock J, Chase J, et al. Pharmacologic inhibition of the Menin-MLL interaction blocks progression of MLL leukemia *in vivo*. Cancer Cell. 2015;27(4):589–602. doi:10.1016/j.ccell.2015.02.016; 25817203 PMC4415852

[37] Aldoss I, Issa GC, Blachly JS, Thirman MJ, Mannis GN, Arellano ML, et al. Updated results and longer follow-up from the AUGMENT-101 phase 2 study of revumenib in all patients with relapsed or refractory (R/R) KMT2Ar acute leukemia. Blood. 2024;144(Suppl 1):211. doi:10.1182/blood-2024-194384.

[38] Issa GC, Cuglievan B, Daver N, DiNardo CD, Farhat A, Short NJ, et al. Phase I/II study of the all-oral combination of revumenib (SNDX-5613) with decitabine/cedazuridine (ASTX727) and venetoclax (SAVE) in R/R AML. Blood. 2024;144(Suppl 1):216. doi:10.1182/blood-2024-204375.38648571

[39] Arellano ML, Thirman MJ, DiPersio JF, Heiblig M, Stein EM, Schuh AC, et al. Menin inhibition with revumenib for *NPM1*-mutated relapsed or refractory acute myeloid leukemia: the AUGMENT-101 study. Blood. 2025;146(9):1065–77. doi:10.1182/blood.2025028357; 40332046 PMC12824668

[40] Issa GC, Cuglievan B, DiPersio JF, Yu L, Jha S, Smith AR, et al. AML-112: revumenib activity in patients with acute leukemia with NUP98r: results from the AUGMENT-101 phase 1 study. Clin Lymphoma Myeloma Leuk. 2025;25:S401–2. doi:10.1016/S2152-2650(25)01624-6.

[41] Zeidner JF, Lin TL, Welkie RL, Curran E, Koenig K, Stock W, et al. Azacitidine, venetoclax, and revumenib for newly Diagnosed NPM1-mutated or *KMT2A*-rearranged AML. J Clin Oncol. 2025;43(23):2606–15. doi:10.1200/jco-25-00914; 40504618 PMC12316144

[42] Wang ES, Issa GC, Erba HP, Altman JK, Montesinos P, DeBotton S, et al. Ziftomenib in relapsed or refractory acute myeloid leukaemia (KOMET-001): a multicentre, open-label, multi-cohort, phase 1 trial. Lancet Oncol. 2024;25(10):1310–24. doi:10.1016/S1470-2045(24)00386-3; 39362248

[43] Wang ES, Montesinos P, Foran J, Erba H, Rodríguez-Arbolí E, Fedorov K, et al. Ziftomenib in relapsed or refractory NPM1-Mutated AML. J Clin Oncol. 2025;43:3381–90; 40997296 10.1200/JCO-25-01694 PMC12573682

[44] Fathi AT, Issa GC, Wang ES, Erba H, Altman JK, Balasubramanian SK, et al. Ziftomenib combined with venetoclax/azacitidine in relapsed/refractory NPM1-m or KMT2A-r acute myeloid leukemia: interim phase 1a results from KOMET-007. Blood. 2024;144(Suppl 1):2880. doi:10.1182/blood-2024-199170.

[45] Zeidan AM, Wang ES, Issa GC, Erba H, Altman JK, Balasubramanian SK, et al. Ziftomenib combined with intensive induction (7+3) in newly diagnosed NPM1-m or KMT2A-r acute myeloid leukemia: interim phase 1a results from KOMET-007. Blood. 2024;144(Suppl 1):214. doi:10.1182/blood-2024-198218.

[46] Wu D, Wang Y, Chen S, Li Y, Huang R, Ren J, et al. A first-in-human phase 1/2 study of the menin-KMT2A(MLL1) inhibitor BN104 in adult patients with relapsed or refractory acute leukemia. Blood. 2024;144(Suppl 1):2879. doi:10.1182/blood-2024-198959.

[47] Searle E, Recher C, Abdul-Hay M, Abedin S, Aldoss I, Pierola AA, et al. Bleximenib dose optimization and determination of RP2D from a phase 1 study in relapsed/refractory acute leukemia patients with KMT2A and NPM1 alterations. Blood. 2024;144(Suppl 1):212. doi:10.1182/blood-2024-207106.

[48] Recher C, O’Nions J, Aldoss I, Pierola AA, Allred A, Alonso-Dominguez JM, et al. Phase 1b study of menin-KMT2A inhibitor bleximenib in combination with intensive chemotherapy in newly diagnosed acute myeloid leukemia with KMT2Ar or NPM1 alterations. Blood. 2024;144(Suppl 1):215. doi:10.1182/blood-2024-207072.

[49] Wei AH, Reyner JE, Garciaz S, Aldoss I, Piérola AA, Allred A, et al. AML-418: recommended phase 2 dose (RP2D) determination of bleximenib in combination with Venetoclax+Azacitidine (VEN+AZA): phase ib study in newly diagnosed (ND) and relapsed/refractory (R/R) acute myeloid leukemia (AML) with KMT2A or NPM1 alterations. Clin Lymphoma Myeloma Leuk. 2025;25:S423–4. doi:10.1016/S2152-2650(25)01665-9.

[50] Lancet J, Ravandi F, Montesinos P, Barrientos JC, Badar T, Alegre A, et al. Covalent menin inhibitor bmf-219 in patients with relapsed or refractory (R/R) acute leukemia (AL): preliminary phase 1 data from the covalent-101 study. Blood. 2023;142(Suppl 1):2916. doi:10.1182/blood-2023-173149.

[51] Zeidner JF, Yuda J, Watts JM, Levis MJ, Erba HP, Fukushima K, et al. Phase 1 results: first-in-human phase 1/2 study of the menin-MLL inhibitor enzomenib (DSP-5336) in patients with relapsed or refractory acute leukemia. Blood. 2024;144(Suppl 1):213. doi:10.1182/blood-2024-194827.

[52] Center for Drug Evaluation and Research. FDA approves revumenib for relapsed or refractory acute myeloid leukemia with a susceptible NPM1 mutation. Silver Spring, MD, USA: U.S. Food and Drug Administration; 2025.

[53] Issa GC, Aldoss I, Thirman MJ, DiPersio J, Arellano M, Blachly JS, et al. Menin inhibition with revumenib for KMT2A-rearranged relapsed or refractory acute leukemia (AUGMENT-101). J Clin Oncol. 2025;43(1):75–84. doi:10.1200/jco.24.00826; 39121437 PMC11687943

[54] Zeidan AM, Fathi AT, Issa GC, Erba HP, Ahsan J, Corum D, et al. Phase 1 study of ziftomenib (KO-539) in combination with venetoclax or venetoclax/azacitidine or standard induction cytarabine/daunorubicin (7+3) chemotherapy for the treatment of patients with acute myeloid leukemia. J Clin Oncol. 2023;41(16 suppl):TPS7079. doi:10.1200/jco.2023.41.16_suppl.tps7079.

[55] Center for Drug Evaluation and Research. FDA approves ziftomenib for relapsed or refractory acute myeloid leukemia with a NPM1 mutation. Silver Spring, MD, USA: U.S. Food and Drug Administration; 2025.

[56] Perner F, Stein EM, Wenge DV, Singh S, Kim J, Apazidis A, et al. MEN1 mutations mediate clinical resistance to menin inhibition. Nature. 2023;615(7954):913–9. doi:10.1038/s41586-023-05755-9; 36922589 PMC10157896

[57] Ray J, Clegg B, Grembecka J, Cierpicki T. Drug-resistant menin variants retain high binding affinity and interactions with MLL1. J Biol Chem. 2024;300(10):107777. doi:10.1016/j.jbc.2024.107777; 39276940 PMC11490872

[58] Zhou X, Zhang L, Aryal S, Veasey V, Tajik A, Restelli C, et al. Epigenetic regulation of noncanonical menin targets modulates menin inhibitor response in acute myeloid leukemia. Blood. 2024;144(19):2018–32. doi:10.1182/blood.2023023644; 39158067 PMC11561541

[59] Wang H, Wang L, Erdjument-Bromage H, Vidal M, Tempst P, Jones RS, et al. Role of histone H2A ubiquitination in Polycomb silencing. Nature. 2004;431(7010):873–8. doi:10.1038/nature02985; 15386022

[60] de Napoles M, Mermoud JE, Wakao R, Tang YA, Endoh M, Appanah R, et al. Polycomb group proteins Ring1A/B link ubiquitylation of histone H2A to heritable gene silencing and X inactivation. Dev Cell. 2004;7(5):663–76. doi:10.1016/j.devcel.2004.10.005; 15525528

[61] Dolnik A, Engelmann JC, Scharfenberger-Schmeer M, Mauch J, Kelkenberg-Schade S, Haldemann B, et al. Commonly altered genomic regions in acute myeloid leukemia are enriched for somatic mutations involved in chromatin remodeling and splicing. Blood. 2012;120(18):e83–92. doi:10.1182/blood-2011-12-401471; 22976956

[62] Hernández-Sánchez A, González T, Sobas M, Sträng E, Castellani G, Abáigar M, et al. Rearrangements involving 11q23.3/KMT2A in adult AML: mutational landscape and prognostic implications—a HARMONY study. Leukemia. 2024;38(9):1929–37. doi:10.1038/s41375-024-02333-4; 38965370 PMC11347382

[63] Bolouri H, Farrar JE, Triche T, Ries RE, Lim EL, Alonzo TA, et al. The molecular landscape of pediatric acute myeloid leukemia reveals recurrent structural alterations and age-specific mutational interactions. Nat Med. 2018;24(1):103–12. doi:10.1038/nm.4439; 29227476 PMC5907936

[64] Yuen KY, Liu Y, Zhou YZ, Wang Y, Zhou DH, Fang JP, et al. Mutational landscape and clinical outcome of pediatric acute myeloid leukemia with 11q23/KMT2A rearrangements. Cancer Med. 2023;12(2):1418–30. doi:10.1002/cam4.5026; 35833755 PMC9883550

[65] Fiskus W, Boettcher S, Daver N, Mill CP, Sasaki K, Birdwell CE, et al. Effective Menin inhibitor-based combinations against AML with MLL rearrangement or NPM1 mutation (NPM1c). Blood Cancer J. 2022;12:5. doi:10.1038/s41408-021-00603-3; 35017466 PMC8752621

[66] Pei S, Pollyea DA, Gustafson A, Stevens BM, Minhajuddin M, Fu R, et al. Monocytic subclones confer resistance to venetoclax-based therapy in patients with acute myeloid leukemia. Cancer Discov. 2020;10(4):536–51. doi:10.1158/2159-8290.CD-19-0710; 31974170 PMC7124979

[67] Kazi JU, Rönnstrand L. FMS-like tyrosine kinase 3/FLT3: from basic science to clinical implications. Physiol Rev. 2019;99(3):1433–66. doi:10.1152/physrev.00029.2018; 31066629

[68] Zhong Y, Qiu RZ, Sun SL, Zhao C, Fan TY, Chen M, et al. Small-molecule fms-like tyrosine kinase 3 inhibitors: an attractive and efficient method for the treatment of acute myeloid leukemia. J Med Chem. 2020;63(21):12403–28. doi:10.1021/acs.jmedchem.0c00696; 32659083

[69] Griffith J, Black J, Faerman C, Swenson L, Wynn M, Lu F, et al. The structural basis for autoinhibition of FLT3 by the juxtamembrane domain. Mol Cell. 2004;13(2):169–78. doi:10.1016/s1097-2765(03)00505-7; 14759363

[70] Levis MJ, Hamadani M, Logan BR, Jones RJ, Singh AK, Litzow MR, et al. Measurable residual disease and posttransplantation gilteritinib maintenance for patients with FLT3-ITD-mutated AML. Blood. 2025;145(19):2138–48. doi:10.1182/blood.2024025154; 39775763 PMC12105721

[71] Yoon JJ, Miao H, Purohit T, Chen D, Cierpicki T, Grembecka J. Combination of menin and IDH mutant inhibition in acute myeloid leukemia. Blood. 2022;140(Suppl 1):5946. doi:10.1182/blood-2022-168834.

[72] Clark O, Yen K, Mellinghoff IK. Molecular pathways: isocitrate dehydrogenase mutations in cancer. Clin Cancer Res. 2016;22(8):1837–42. doi:10.1158/1078-0432.CCR-13-1333; 26819452 PMC4834266

[73] Sparbier CE, Gillespie A, Gomez J, Kumari N, Motazedian A, Chan KL, et al. Targeting Menin disrupts the KMT2*A*/B and polycomb balance to paradoxically activate bivalent genes. Nat Cell Biol. 2023;25(2):258–72. doi:10.1038/s41556-022-01056-x; 36635503 PMC7614190

[74] Liu S, Hu W, Yang D, Han L, Tong M, Yan Y, et al. Discovery of BTC-86, a novel second-generation menin-MLL inhibitor to overcome the acquired resistance in MEN1 for R/R acute leukemia. Blood. 2024;144(Suppl 1):3586. doi:10.1182/blood-2024-202042.

[75] Nadiminti KVG, Sahasrabudhe KD, Liu H. Menin inhibitors for the treatment of acute myeloid leukemia: challenges and opportunities ahead. J Hematol Oncol. 2024;17(1):113. doi:10.1186/s13045-024-01632-8; 39558390 PMC11575055

[76] Di Fazio P. Targeting menin: a promising therapeutic strategy for susceptible acute leukemia subtypes. Signal Transduct Target Ther. 2023;8(1):384. doi:10.1038/s41392-023-01627-w; 37816719 PMC10564860

[77] Tiong IS, Ritchie DS, Blombery P. Response and resistance to menin inhibitor in UBTF-tandem duplication AML. N Engl J Med. 2024;390(24):2323–5. doi:10.1056/NEJMc2404110; 38924737

[78] Li Q, Xing S, Zhang H, Mao X, Xiao M, Wang Y. FISH improves risk stratification in acute leukemia by identifying KMT2A abnormal copy number and rearrangements. Sci Rep. 2022;12(1):9585. doi:10.1038/s41598-022-13545-y; 35688861 PMC9187764

[79] Döhner H, Wei AH, Appelbaum FR, Craddock C, DiNardo CD, Dombret H, et al. Diagnosis and management of AML in adults: 2022 recommendations from an international expert panel on behalf of the ELN. Blood. 2022;140(12):1345–77. doi:10.1182/blood.2022016867; 35797463

[80] Bourgeois W, Cutler J, Rice HE, Regalado B, Wenge DV, Perner F, et al. Discerning the landscape of menin inhibitor resistance. Blood. 2024;144(Suppl 1):724. doi:10.1182/blood-2024-208887.

[81] D’Souza J, Leung CJ, Ballapuram AC, Lin AS, Batingana AR, Lamble AJ, et al. TP53 inactivation confers resistance to the menin inhibitor revumenib in acute myeloid leukemia. BioRxiv:644993. 2025. doi:10.1101/2025.03.24.644993.

[82] Kim K, Maiti A, Loghavi S, Pourebrahim R, Kadia TM, Rausch CR, et al. Outcomes of TP53-mutant acute myeloid leukemia with decitabine and venetoclax. Cancer. 2021;127(20):3772–81. doi:10.1002/cncr.33689; 34255353 PMC10462434

[83] Zucenka A, Issa GC, Arellano M, Khazal S, Khera N, Stock W, et al. Revumenib maintenance therapy following revumenib-induced remission and transplant. Blood. 2023;142(Suppl 1):4950. doi:10.1182/blood-2023-189036.

